# Environmental assessment of cytotoxic drugs in healthcare settings: protocol for a systematic review and meta-analysis

**DOI:** 10.1186/s13643-020-01494-4

**Published:** 2020-10-19

**Authors:** Laila Al Alawi, Elpidoforos S. Soteriades, Marilia Silva Paulo, Linda Östlundh, Michal Grivna, Fatima Al Maskari, Rami H. Al-Rifai

**Affiliations:** 1 Institute of Public Health, College of Medicine and Health Sciences, United Arab Emirates University, P.O. Box 17666, Al Ain, United Arab Emirates; 2 Department of Environmental Health, Environmental and Occupational Medicine and Epidemiology (EOME), Harvard School of Public Health, Boston, MA USA; 3 Global Health and Tropical Medicine, Instituto de Higiene e Medicina Tropical, Universidade Nova de Lisboa, Lisbon, Portugal; 4 National Medical Library, College of Medicine and Health Sciences, United Arab Emirates University, Al Ain, United Arab Emirates; 5 Zayed Center for Health Sciences, United Arab Emirates University, Al Ain, United Arab Emirates

**Keywords:** Cytotoxic drugs, Environmental assessment, Systematic review, Meta-analysis

## Abstract

**Background:**

Occupational exposure to cytotoxic drugs is associated with various unfavorable health outcomes. This protocol reports a methodology for a systematic review and meta-analysis that aims to systematically review the published literature and quantify the level of environmental contamination of healthcare settings with cytotoxic drugs.

**Methods:**

This protocol is developed in accordance with the Preferred Reporting Items for Systematic Reviews and Meta-Analyses Protocol-2015 (PRISMA-P) guidelines. Six electronic databases (PubMed, Web of Science, Scopus, Cochrane Library, CINAHL, and EMBASE) will be searched with no restrictions on publication period. Eligible studies will be identified and data will be extracted using a predefined data extraction form by at least two independent reviewers following best practice. Eligible studies should report calculated or calculable estimates on the proportion of positive samples tested for cytotoxic drugs and/or estimates on the concentration of the cytotoxic drug(s) in the tested samples. Risk of bias (RoB) will be assessed by using the RoB in Studies estimating Prevalence of Exposure to Occupational risk factors (RoB-SPEO) tool, which developed by the World Health Organization (WHO) and International Labour Organization (ILO) for environmental and occupational health systematic reviews. The random-effects model will be used to perform meta-analyses.

**Discussion:**

Occupational exposure to cytotoxic drugs is associated with short- and long-term adverse health outcomes. Following this protocol, the review to be carried out will be the first to fill an evidence gap on the environmental contamination of healthcare settings with cytotoxic drugs. The findings of this review will help in the understanding of the risk of occupational exposure of healthcare workers to cytotoxic drugs and facilitate the identification of priority areas for specific interventions.

**Ethics and dissemination:**

The systematic review methodology does not require ethics approval due to the nature of the study design. The results of the systematic review will be published in a peer-reviewed journal and will be publicly available.

**Systematic review registration:**

PROSPERO CRD42020162780, dated July 14, 2020

## Background

Hazardous drugs, as defined by the National Institute for Occupational Safety and Health (NIOSH), are the drugs that associated with genotoxicity, carcinogenicity, teratogenicity, reproductive toxicity, organ toxicity, or other developmental toxicity [1]. Cytotoxic drugs, also known as anti-neoplastic, chemotherapeutic, or hazardous drugs [2], are defined as a group of medicines designed to treat cancer through disrupting the cell cycle and destroy cells that grow in a rapid and uncontrolled manner [3]. They have extensively been used in the treatment of cancer patients and this has led to occupational hazards associated with exposure of healthcare professionals to such drugs leading to different adverse effects including spontaneous abortion [4], infertility [5, 6], premature labor, developmental and behavioral abnormalities among children of exposed workers, and potential cancer development [6]. Furthermore, several studies have reported widespread contamination of working areas which leads to healthcare workers’ exposure to these hazardous drugs [7, 8].

Potential routes of occupational exposure may include dermal absorption, ingestion, and inhalation [1]. While levels of such exposures are usually much lower than those administered to cancer patients [4], occupational exposure likely involves a concurrent exposure to more than one cytotoxic drugs or several combined exposures, and it occurs more frequently over a much longer period of time leading to mixed cumulative exposures [9]. Furthermore, occupational exposures are often unrecognized due to lack of systematic environmental assessment and biomonitoring programs in oncology departments [10].

A recent systematic review conducted by the National Toxicology Program (NTP) USA 2019 [9] evaluated the literature on occupational exposure to cytotoxic drugs and its associated adverse health effects in humans. This systematic review also summarized the prevalence and levels of cytotoxic drugs detected in the workplace. According to this review, detectable levels of cytotoxic drugs continue to be identified in the workplace environment [9]. However, the risk of bias (RoB) of the reviewed studies and evaluation of confidence in the body of evidence were not reported. In addition, the review did not quantify the levels of environmental contamination of the workplace with cytotoxic drugs [9].

Several individual studies have been conducted on the cytotoxic drugs contamination in healthcare settings. These individual studies do not however assess the worldwide burden of cytotoxic drugs contamination in healthcare settings. Furthermore, the evidence is lacking on pooled estimates of the levels of healthcare setting contamination in order to provide national, international, and global figures. Therefore, the proposed systematic review and meta-analysis following this protocol aims to (1) systematically review the published literature, (2) estimate the pooled prevalence of environmental samples positive to cytotoxic drugs, and (3) estimate the weighted mean concentration of cytotoxic drugs, in the environmental samples collected from different healthcare settings around the world.

## Methods and data synthesis

The review to be carried out following this protocol will be reported in accordance to the Preferred Reporting Items for Systematic Reviews and Meta-analyses (PRISMA) guidelines [11].

### Outcome

The main outcome measure in our study will be the pooled prevalence of positive samples of cytotoxic drugs in the tested environmental samples, and the mean concentration of cytotoxic drugs in environmental samples collected from healthcare settings.

### Protocol design and registration

The development of this protocol is in accordance with the Preferred Reporting Items for Systematic Reviews and Meta-analyses Protocol (PRISMA-P) guidelines (S1 Table) [12]. The protocol is registered (ID CRD42020162780) on The International Prospective Register of Systematic Reviews (PROSPERO). Registration reduces duplication of reviews and provides transparency in the review process, with the aim of minimizing reporting bias. In the S2 Table, we provide an indicative International Agency for Research on Cancer (IARC) classification of cytotoxic drugs that have been adopted and will be used in our planned review [13, 14].

### Eligibility criteria

We will include studies conducted in healthcare settings with no restrictions on publication or study period. All studies including samples from settings involved in the preparation, transport, administration, and waste disposal of cytotoxic drugs, will be considered. We will also consider healthcare settings in which these settings are exposed to cytotoxic drugs as participants with regard to outcomes such as surface contamination and aerosol contamination. Eligible studies should report quantitative data on the prevalence of positive samples and/or on the concentration of the detected cytotoxic drugs in the positive samples. A positive sample would be defined as a sample with cytotoxic drugs above the level of detection limit of cytotoxic drugs [15]. Studies reporting on calculated prevalence or those presenting data that would allow us to calculate the prevalence or concentration of cytotoxic drugs in the collected environmental samples will be included. No restrictions on study design will be applied. We will include only articles reported in the English language.

Studies will be excluded if are not conducted in healthcare settings such as those conducted in university laboratories, drug manufacturing companies and/or veterinaries. Studies that do not provide quantitative information on the prevalence or the concentration of positive environmental samples of cytotoxic drug residues will be not be deemed eligible for inclusion.

### Search strategy and searching sources

A comprehensive systematic search in PubMed, Web of Science (Core Collection), Scopus, Cochrane Library, CINAHL, and Embase and in sources for grey literature will be conducted. The search strategy will be developed by the project team under the guidance of an experienced librarian. PubMed and PubMed’s MeSH will be used to systematically develop a comprehensive search string. All search terms will be searched in a combination of title, abstract, and MeSH/Thesaurus (when available) to ensure the best possible information retrieval. A filter for the English language will be applied. All publication years and publication types will be included. A detailed search log with transparent and reproducible search stings, results, and search variation notes for all included databases and sources for grey literature will be developed and reported. A preliminary search strategy conducted in PubMed is available in the S3 Table. Hand searching of the reference lists for studies that might have been potentially missed will be conducted.

### Study selection

All citations identified through the literature search will be imported into the systematic review software “Covidence” [16] for deduplication and blinded screening. Titles and abstracts of the retrieved citations will be screened to exclude all ineligible studies against the pre-set inclusion criteria. Full texts of the eligible and potentially eligible studies will be thoroughly assessed for eligibility by at least two independent reviewers. Disagreements will be resolved through discussion and consensus after consulting a third reviewer whenever necessary. The corresponding authors of eligible articles will be contacted for clarification whenever needed. We will record all reasons for the exclusion and report the study selection process using the PRISMA flow diagram [11].

### Data extraction

For studies found eligible to be included in the systematic review, relevant data will be extracted into a predefined data extraction form, which will first be piloted using five eligible studies. Data will be extracted by at least two reviewers. Data to be extracted from each eligible study will include baseline and methodological data. We will extract information related to the authors’ names, publication year, country, studied cytotoxic drugs, sample size, sampling locations, sampling year(s), analytical tool, the sensitivity of contaminant measurements [limit of detection (LOD) or limit of quantitation (LOQ)] [17], number of tested samples, number of positive samples, and mean concentration of the tested cytotoxic drugs in the tested samples. A list of variables to be extracted from eligible studies are provided in S4 Table.

### Quality and risk of bias assessment

At least two reviewers will independently evaluate and assess the methodological quality of the eligible studies. If required, the authors of the studies will be contacted to request missing or additional data for an explanation. Disagreements between the reviewers will be resolved by discourse. The results will be reported in narrative form and summarized in tables.

The risk of bias (RoB) of the included studies will be assessed using the RoB in Studies estimating Prevalence of Exposure to Occupational risk factors (RoB-SPEO) tool developed by the World Health Organization (WHO) and the International Labour Organization (ILO) for studies of the prevalence of exposure to occupational risk factors [18]. We will assess the RoB on the levels of each individual study and the entire body of evidence overall. We will resolve any disagreements by discussion. The RoB will be assessed according to the following domains: (i) selection bias, (ii) performance bias, (iii) misclassification bias, (iv) conflict of interest, and (v) other biases. Categorization of bias will be “low,” “probably low,” “probably high,” “high,” or “not applicable.”

### Quality of evidence

The quality of evidence of the included studies will be assessed using the quality and strength of evidence ratings proposed by the Navigation Guide as a framework. We will decrease, or not, the quality level of the body of evidence based on the (i) RoB across studies, (ii) indirectness of the evidence, (iii) inconsistency, (iv) imprecision, and (v) publication bias [19, 20]. We will grade the evidence, using the three Navigation Guide quality of evidence ratings: “high,” “moderate,” and “low” [20]. Within each of the relevant reasons for downgrading, we will rate any concern per reason as “none,” “serious,” or “very serious.”

## Synthesis of evidence: meta-analysis

### Pooled weighted measures

We will provide a quantitative synthesis approach to report the prevalence of cytotoxic drugs contamination in environmental samples collected from healthcare settings. To estimate the pooled prevalence, we will conduct a meta-analysis using a random-effects model to estimate the pooled prevalence of positive samples for the tested cytotoxic contamination. We will use the *metaprop* command to perform a meta-analysis of the prevalence estimates [21]. To estimate the mean concentration of cytotoxic drugs in the tested environmental samples in healthcare settings, we will conduct a meta-analysis using a random-effects model. We will use the *metan* command to perform meta-analysis of cytotoxic drugs concentration in the tested samples. The pooled measures will be weighted using the inverse variance method [22]. For each pooled estimate and its 95% confidence interval (CI), a forest plot will be created to show the estimated overall weighted prevalence/weighted mean concentration and its corresponding 95% CI for each study following the Cochrane guidelines [23].

Heterogeneity between the studies in effect measures will be assessed using both the *Q*-test statistic and the *I*-squared (*I*^2^) statistic. We will consider a *Q*-test with a *p* -value < 0.05 and *I*^2^ statistic of > 50% as indicative of substantial heterogeneity. 

### Subgroup analyses

Depending on data availability, we expect to conduct subgroup analyses stratifying by geographical regions, time periods, type of the tested cytotoxic drug, study settings, sample locations, and quality/bias assessment classifications. We will also conduct subgroup analyses based on study quality.

## Discussion

The extensive use of cytotoxic drugs in the treatment of cancer patients has led to occupational hazards associated with exposure of healthcare professionals to such drugs. Exposure to cytotoxic drugs leads to different adverse health effects such as spontaneous abortion, infertility, premature labor, and developmental and behavioral abnormalities. Systematically compiling and summarizing primary studies as well as providing comprehensive estimates on the burden of cytotoxic drugs in healthcare settings is still lacking in the literature. This protocol reduces the possibility of duplication, minimizes possible biases, and allows peer-review using the proposed methods to conduct a high-quality systematic review and meta-analysis. The systematic review to be produced following this protocol, as far as we know, is the first systematic review and meta-analysis to provide systematic evidence on the levels of environmental contamination of the healthcare settings with cytotoxic drugs. The results of the systematic review will be published in a peer-reviewed journal and will be publicly available. The findings will also be disseminated electronically and in printed versions.

## Supplementary information

**Additional file 1: Table S1.** PRISMA-P (Preferred Reporting Items for Systematic review and Meta-Analysis Protocols) 2015 checklist.

**Additional file 2: Table S2.** IARC classification of cytotoxic drugs.

**Additional file 3: Table S1.** Search specifications: All terms are searched in the fields for “Title” and “Abstract” and in “MeSH” when available. No filters or limitations applied .

**Additional file 4: Table S1.** List of string and numerical variables to be extracted from eligible studies.

## Data Availability

Data sharing is not applicable to this article as no datasets were generated or analyzed during the current study.

## Abbreviations

CI: Confidence interval
IARC: International Agency for Research on Cancer
ILO: International Labour Organization
*I*^2^: I-Squared
LOD: Limit of detection
LOQ: Limit of quantitation
NIOSH: National Institute for Occupational Safety and Health
NTP: National Toxicology Program
PRISMA-P: Preferred Reporting Items for Systematic Reviews and Meta-analyses Protocol
PRISMA: Preferred Reporting Items for Systematic Reviews and Meta-analyses
PROSPERO: International Prospective Register of Systematic Review
RoB-SPEO: Risk of Bias in Studies estimating Prevalence of Exposure to Occupational risk factors
WHO: World Health Organization
