# Supplementary material for: Environmental assessment of cytotoxic drugs in healthcare settings: protocol for a systematic review and meta-analysis
Source: Syst Rev. 2020 Oct 19;9:242. doi: 10.1186/s13643-020-01494-4 (PMC7574301; doi:10.1186/s13643-020-01494-4)
Supplement: Supplementary file 2 — Additional file 2: Table S2. IARC classification of cytotoxic drugs. [file 13643_2020_1494_MOESM2_ESM.docx]

**S2 Table.** IARC Classification of cytotoxic drugs [13, 14]

| **Group 1 (Carcinogenic to humans)** | **Group 2A (Probably carcinogenic to humans)** | **Group 2B (Possibly carcinogenic to humans)** | **Group 3 (Not classified as to carcinogenicity in humans)** |
| --- | --- | --- | --- |
| Azathioprine | Azacitidine | Bleomycins | 5-Fluorouracil |
| N,N-Bis(2-chloroethyl)-2-naphthylamine ( Chlornaphazine) | Bischloroethyl nitrosourea (BCNU) | Dacarbazine | Isophosphamide |
| 1,4-Butanediol dimethanesulfonate (Busulfan; Myleran) | Cisplatin | Daunomycin | 6-Mercaptopurine |
| Chlorambucil | 1-(2-Chloroethyl)-3-cyclohexyl-1-nitrosourea (CCNU) (Lomustine) | Mitomycin C | Methotrexate |
| Semustine[1-(2-Chloroethyl)-3-(4-methylcyclohexyl)-1-nitosourea (Methyl-CCNU] | N-Methyl-N-nitrosourea | Mitoxantrone | Prednisone |
| Cyclophosphamide | N-Ethyl-N-nitrosourea | Streptozotocin | Vinblastine sulfate |
| Etoposide | Nitrogen mustard ( Mechlorethamine) | Merphalan | Vincristine sulfate |
| Etoposide in combination with cisplatin and bleomycin | Procarbazine hydrochloride | Amsacrine |  |
| Melphalan | Teniposide (Vumon) | Aziridine |  |
| MOPP and other combined chemotherapy including alkylating agents | Adriamycin |  |  |
| Thiotepa | Chlorozotocin |  |  |
| Treosulfan |  |  |  |
| Tamoxifen |  |  |  |

1. International Agency for Research on Cancer (IARC), Agents Classified by the IARC Monographs, List of Classifications.

Volumes 1-125. World Health Organization. 2019. Available at https://dtsc.ca.gov/wp-content/uploads/sites/31/2019/05/1-J-IARC-carcin.pdf.

1. Fransman W: Antineoplastic drugs: Occupational exposure and health risks. Utrecht University Repository. Ridderprint offsetdrukkerij BV, Ridderkerk, the Netherlands; 2006:12. Available from: https://dspace.library.uu.nl/handle/1874/12854.
