# Supplementary material for: Environmental assessment of cytotoxic drugs in healthcare settings: protocol for a systematic review and meta-analysis
Source: Syst Rev. 2020 Oct 19;9:242. doi: 10.1186/s13643-020-01494-4 (PMC7574301; doi:10.1186/s13643-020-01494-4)
Supplement: Supplementary file 3 — Additional file 3: Table S1. Search specifications: All terms are searched in the fields for “Title” and “Abstract” and in “MeSH” when available. No filters or limitations applied . [file 13643_2020_1494_MOESM3_ESM.docx]

**S3 Table.** Pre-search in PubMed, 2020-08-20

**Search specifications:** All terms are searched in the fields for “Title” and “Abstract” and in “MeSH” when available. No filters or limitations applied.

### Result: 983 references

| **Search string** |
| --- |
| (("Hospitals"[Mesh] OR "Oncology Nursing"[Mesh] OR "Oncology Service, Hospital"[Mesh] OR "Medical Oncology"[Mesh] "Adult Day Care Centers"[Mesh] OR "Health Facilities"[Mesh] OR "cancer care facilities" [Title/Abstract] OR "cancer care facility"[Title/Abstract] OR "outpatient clinic" [Title/Abstract] OR "outpatient clinics" [Title/Abstract] OR "healthcare environment"[Title/Abstract] OR "work environment"[Title/Abstract] OR "workplace environment"[Title/Abstract] OR hospital[Title/Abstract] OR hospitals[Title/Abstract] OR "oncology"[Title/Abstract] OR "day care centres"[Title/Abstract] OR "day care centers"[Title/Abstract] OR "day care centre"[Title/Abstract] OR "day care center"[Title/Abstract] OR "medical centres"[Title/Abstract] OR "medical centers"[Title/Abstract] OR "pharmacy"[Title/Abstract] OR "pharmacies"[Title/Abstract] OR "patient care area"[Title/Abstract] OR "patient care areas"[Title/Abstract] OR "inpatient ward"[Title/Abstract] OR "outpatient ward"[Title/Abstract] OR "preparation room"[Title/Abstract] OR "administration room"[Title/Abstract] OR "healthcare facilities"[Title/Abstract] OR "health facility"[Title/Abstract] OR "medical facility"[Title/Abstract] OR "medical facilities"[Title/Abstract] OR "Health Personnel"[Mesh] OR "health care professionals"[Title/Abstract] OR "health personnel"[Title/Abstract] OR "health care providers"[Title/Abstract] OR "health care provider"[Title/Abstract] OR "healthcare providers"[Title/Abstract] OR "healthcare workers"[Title/Abstract] OR "healthcare worker"[Title/Abstract] OR "healthcare professionals"[Title/Abstract] OR "health care professional"[Title/Abstract] OR "healthcare professional"[Title/Abstract] OR "healthcare personnel"[Title/Abstract] OR "health care personnel"[Title/Abstract] OR "health care workers"[Title/Abstract] OR "medical worker"[Title/Abstract] OR "healthcare staff"[Title/Abstract] OR "health care staff"[Title/Abstract] OR "pharmacists"[Title/Abstract] OR "pharmacy technicians"[Title/Abstract] OR "nurses"[Title/Abstract] OR "nurse"[Title/Abstract] OR "drug-handlers"[Title/Abstract] OR physician*[Title/Abstract] OR doctor*[Title/Abstract] OR "physician assistants" [Title/Abstract] OR "physicians' assistants" OR "physician assistant" [Title/Abstract] OR "physicians' assistant" OR "doctor's assistants" OR "doctor assistants" OR "doctor's assistant" OR "doctor assistant" OR "physicians' extenders" OR "physicians extenders" OR "physicians' extender" OR "physicians extender" OR clinician*[Title/Abstract] OR "healthcare technicians"[Title/Abstract] OR "healthcare technician"[Title/Abstract] OR "medical technicians"[Title/Abstract] OR "medical technician"[Title/Abstract]) AND ("Equipment Contamination"[Mesh] OR "equipment contamination"[Title/Abstract] OR "environmental assessment"[Title/Abstract] OR "environmental assessments"[Title/Abstract] OR "surface contamination"[Title/Abstract] OR "surface contaminations"[Title/Abstract] OR "exposure assessment"[Title/Abstract] OR "exposure assessments"[Title/Abstract] OR "personal protective equipment"[Title/Abstract] OR "biological safety cabinet"[Title/Abstract] OR "BSC"[Title/Abstract] OR "closed system devices"[Title/Abstract] OR "closed-system transfer device"[Title/Abstract] OR "closed-system drug transfer device"[Title/Abstract] OR "drug transfer device"[Title/Abstract] OR "robotic system"[Title/Abstract] OR "robotic systems"[Title/Abstract] OR "compounding aseptic containment isolator"[Title/Abstract] OR "environmental sampling"[Title/Abstract] OR "environmental monitoring"[Title/Abstract] OR "environmental contamination"[Title/Abstract] OR "workplace contamination"[Title/Abstract] OR "contamination levels"[Title/Abstract] OR "contamination level"[Title/Abstract] OR "wipe samples"[Title/Abstract] OR "wipe sampling"[Title/Abstract] OR "environmental exposure"[Title/Abstract] OR "workplace exposure"[Title/Abstract] OR "workers exposure"[Title/Abstract] OR "control measures"[Title/Abstract] OR "occupational risk"[Title/Abstract] OR "occupational risks"[Title/Abstract] OR "occupational hazards"[Title/Abstract] OR "occupational hazard"[Title/Abstract] OR "occupational exposure"[Title/Abstract] OR "Occupational Exposure"[Mesh] OR "occupational exposures"[Title/Abstract] OR "occupational medicine"[Title/Abstract] OR "Occupational Medicine"[Mesh] OR "employee health"[Title/Abstract] OR "Personal Protective Equipment"[Mesh] OR "personal protective equipment"[Title/Abstract] OR "personal protective equipments"[Title/Abstract] OR "PPE" [Title/Abstract])) AND ("antitumor drugs"[Title/Abstract] OR "antitumor drug"[Title/Abstract] OR "antitumor agent"[Title/Abstract] OR "antitumor agents"[Title/Abstract] OR "cytotoxic drug"[Title/Abstract] OR "cytotoxic drugs"[Title/Abstract] OR "cytotoxic agent"[Title/Abstract] OR "cytotoxic agents"[Title/Abstract] OR "cytostatic drugs"[Title/Abstract] OR "cytostatic drug"[Title/Abstract] OR "cytostatic agent"[Title/Abstract] OR "cytostatic agents"[Title/Abstract] OR "Cytostatic Agents"[Mesh] OR "antineoplastic drugs"[Title/Abstract] OR "antineoplastic drug"[Title/Abstract] OR "antineoplastic agent"[Title/Abstract] OR "antineoplastic agents"[Title/Abstract] OR "Antineoplastic Agents"[Mesh] OR "hazardous drugs"[Title/Abstract] OR "hazardous drug"[Title/Abstract] OR "anticancer drugs"[Title/Abstract] OR "anticancer drug"[Title/Abstract] OR "anticancer agent"[Title/Abstract] OR "anticancer agents"[Title/Abstract] OR "chemotherapy drugs"[Title/Abstract] OR "chemotherapy drug"[Title/Abstract] OR "chemotherapy agent"[Title/Abstract] OR "chemotherapy agents"[Title/Abstract] OR "chemotherapeutic drugs"[Title/Abstract] OR "chemotherapeutic drug"[Title/Abstract] OR "chemotherapeutic agent"[Title/Abstract] OR "chemotherapeutic agents"[Title/Abstract] OR "cancer drugs"[Title/Abstract] OR "cancer drug"[Title/Abstract] OR "anti-carcinogenic"[Title/Abstract] OR "anticarcinogenic drug"[Title/Abstract] OR "anticarcinogenic drugs"[Title/Abstract] OR "anticarcinogenic agent"[Title/Abstract] OR "anticarcinogenic agents"[Title/Abstract] OR "Anticarcinogenic Agents"[Mesh] OR docetaxel[Title/Abstract] OR paclitaxel[Title/Abstract] OR etoposide[Title/Abstract] OR gemcitabine[Title/Abstract] OR cytarabine[Title/Abstract] OR doxorubicin[Title/Abstract] OR methotrexate[Title/Abstract] OR "5-fluorouracil"[Title/Abstract] OR ifosfamide[Title/Abstract] OR cyclophosphamide[Title/Abstract] OR "Docetaxel"[Mesh] OR "Paclitaxel"[Mesh] OR "Etoposide"[Mesh] OR "Cytarabine"[Mesh] OR "Doxorubicin"[Mesh] OR "Methotrexate"[Mesh] OR "Ifosfamide"[Mesh] OR "Cyclophosphamide"[Mesh] OR "hazardous agent"[Title/Abstract] OR "hazardous agents"[Title/Abstract] OR "chemotherapies"[Title/Abstract] OR "anti-tumor"[Title/Abstract] OR "category-D"[Title/Abstract] OR "category-X"[Title/Abstract] OR "oncology drugs"[Title/Abstract]) |
