# Supplementary material for: Environmental assessment of cytotoxic drugs in healthcare settings: protocol for a systematic review and meta-analysis
Source: Syst Rev. 2020 Oct 19;9:242. doi: 10.1186/s13643-020-01494-4 (PMC7574301; doi:10.1186/s13643-020-01494-4)
Supplement: Supplementary file 4 — Additional file 4: Table S1. List of string and numerical variables to be extracted from eligible studies. [file 13643_2020_1494_MOESM4_ESM.docx]

**S4 Table.** String and numerical variables to be extracted from eligible studies

| **Variable Name** | **Variable Type** | **Variable Label** | **Values** |
| --- | --- | --- | --- |
|  |  |  |  |
| Record | Numeric | Study record number | Numeric |
| Author | String | First author’s name, initials, et al | String |
| Pub_year | Numeric | Publication year | Numeric |
| Journal | String | Journal where the study published | String |
| Country | String | Country where the study was executed | String |
| City | String | City where the study was executed | String |
| Study_des | Numeric | Study design | 1   Cross-sectional |
|  |  |  | 2   Prospective cohort |
|  |  |  | 3   Retrospective cohort |
|  |  |  | 4   Case-control |
|  |  |  | 5   Unclear |
| Yrstart | Date (MM/DD/YYYY) | Year data collection started | Numeric |
| Yrend | Date (MM/DD/YYYY) | Year data collection ended | Numeric |
| Duration | Numeric | Data collection duration in years | Numeric |
| Location | Numeric | Location from where the environmental sample were collected | 1 Pharmacy |
|  |  |  | 2 Store |
|  |  |  | 3 Inpatient ward |
|  |  |  | 4 Laboratory |
|  |  |  | 5 Outpatient clinic |
|  |  |  | 6 …………………… |
|  |  |  | 4 …………………… |
|  |  |  | 5…………………… |
|  |  |  | 6 …………………… |
|  |  |  | 7 …………………… |
| Sampling_strategy | Numeric | Subjects sampling strategy | 1  Convenience |
|  |  |  | 2  Systematic |
|  |  |  | 3  Consecutive |
|  |  |  | 4  Random sampling |
|  |  |  | 5  Multistage probability sampling |
|  |  |  | 6   Unclear |
| Cyto_drug | Numeric | Tested cytotoxic drugs | Name of the tested cytotoxic drug |
| Anal_tool | String | Analytical tool [High-performance liquid chromatography-tandem mass spectroscopy (LC-MS/MS) or Inductively coupled plasma mass spectrometry (ICP-Ms)] | 1 LC-MS/MS  2 ICP-Ms |
| Sensitivity | Numeric | Sensitivity of contaminant measurements | 1 LOD  2 LOQ |
| Sample | Numeric | Tested sample size | Numeric |
| Positive | Numeric | Number of the positive sample | Numeric |
| Mean_Con | Numeric | Mean concentration of the tested cytotoxic drug | Numeric |
| Stdev | Numeric | Standard deviation of the mean concentration of cytotoxic drugs in the tested sample | Numeric |
| Unit | String | Unite of the measured mean concentration (e.g. nanogram) | String |

LC-MS/MS: High-Performance Liquid Chromatography-tandem Mass Spectroscopy

ICP-Ms: Inductively Coupled Plasma-Mass Spectrometry

LOD: Limit of Detection

LOQ: Limit of Quantitation
